# Animal models of cancer metastasis to the bone

**DOI:** 10.3389/fonc.2023.1165380

**Published:** 2023-04-05

**Authors:** Yihan Yu, Kanglu Li, Yizhong Peng, Wei Wu, Fengxia Chen, Zengwu Shao, Zhicai Zhang

**Affiliations:** ^1^ Department of Orthopedics, Union Hospital, Tongji Medical College, Huazhong University of Science and Technology, Wuhan, Hubei, China; ^2^ Department of Radiation and Medical Oncology, Zhongnan Hospital, Wuhan University, Wuhan, Hubei, China

**Keywords:** bone metastases, animal models, breast cancer, prostate cancer, cell lines

## Abstract

Cancer metastasis is a major cause of mortality from several tumors, including those of the breast, prostate, and the thyroid gland. Since bone tissue is one of the most common sites of metastasis, the treatment of bone metastases is crucial for the cure of cancer. Hence, disease models must be developed to understand the process of bone metastasis in order to devise therapies for it. Several translational models of different bone metastatic tumors have been developed, including animal models, cell line injection models, bone implant models, and patient-derived xenograft models. However, a compendium on different bone metastatic cancers is currently not available. Here, we have compiled several animal models derived from current experiments on bone metastasis, mostly involving breast and prostate cancer, to improve the development of preclinical models and promote the treatment of bone metastasis.

## 1 Introduction

Metastasis is a frequent malignant manifestation of cancer in the mid to late stages of tumor progression. Metastasis to the bone, one of the most common sites, occurs when cancer cells migrate from the original site and invade bone tissue. It indicates adverse prognosis, and can cause severe pain, fractures, impaired mobility, and death. The invasion of cancer cells into target sites involves several stages. Initially, they invade the surroundings of the original site, breaching the vasculature and entering the circulation. Then, depending on molecular signals on cell membranes or in their microenvironment, they invade a particular target organ along their path of circulation (1, 2). Although the precise process has not been elucidated yet, the invasion appears to last many months if not years (3). Once a bulk of invasive cancer cells agglomerate into a mass, metastasis begins. Cancer cells modify the surrounding tissues and vasculature to favor their growth. Cancer treatment often involves a combination of radiation, chemotherapy, and medications to reduce the pain and inflammation.

Breast cancer, one of the most prevalent malignant tumors, exhibits a 40% likelihood to eventually develop bone metastases (4, 5). Bone tissue is the most common target site of breast cancer. Bone metastasis reflects potential skeletal-related events and poor clinical results. To improve the current therapies for bone-metastasized breast cancer, animal models that mimic the human tumor microenvironment have been used in preclinical experiments (6). Prostate cancer is the second most frequently occurring cancer in men. It preferentially metastasizes to the bone, and presents a worse prognosis at the metastatic stage. Rarely lethal when restricted to its primary site, the 5-year-survival rate of prostate cancer decreases by 29.8% when it metastasizes to the bone, explaining its rank as the fifth leading cause of tumor-related mortality in males (7). Antimetastatic agents need to be urgently developed and the prognosis following bone metastasis must be improved.

Multiple animal models have been used in clinical research to explore the mechanisms and prognosis of tumor metastasis. Translational models have been used to study the advanced stages of tumor metastases, reveal potential protein targets, and develop metastasis-related treatments. However, fully reproducing human bone metastases in animal models is difficult. Nevertheless, by selecting different cell lines, animal strains, and tumor transplantation methods, animal models can be constructed to answer various questions.

**Figure 1:**
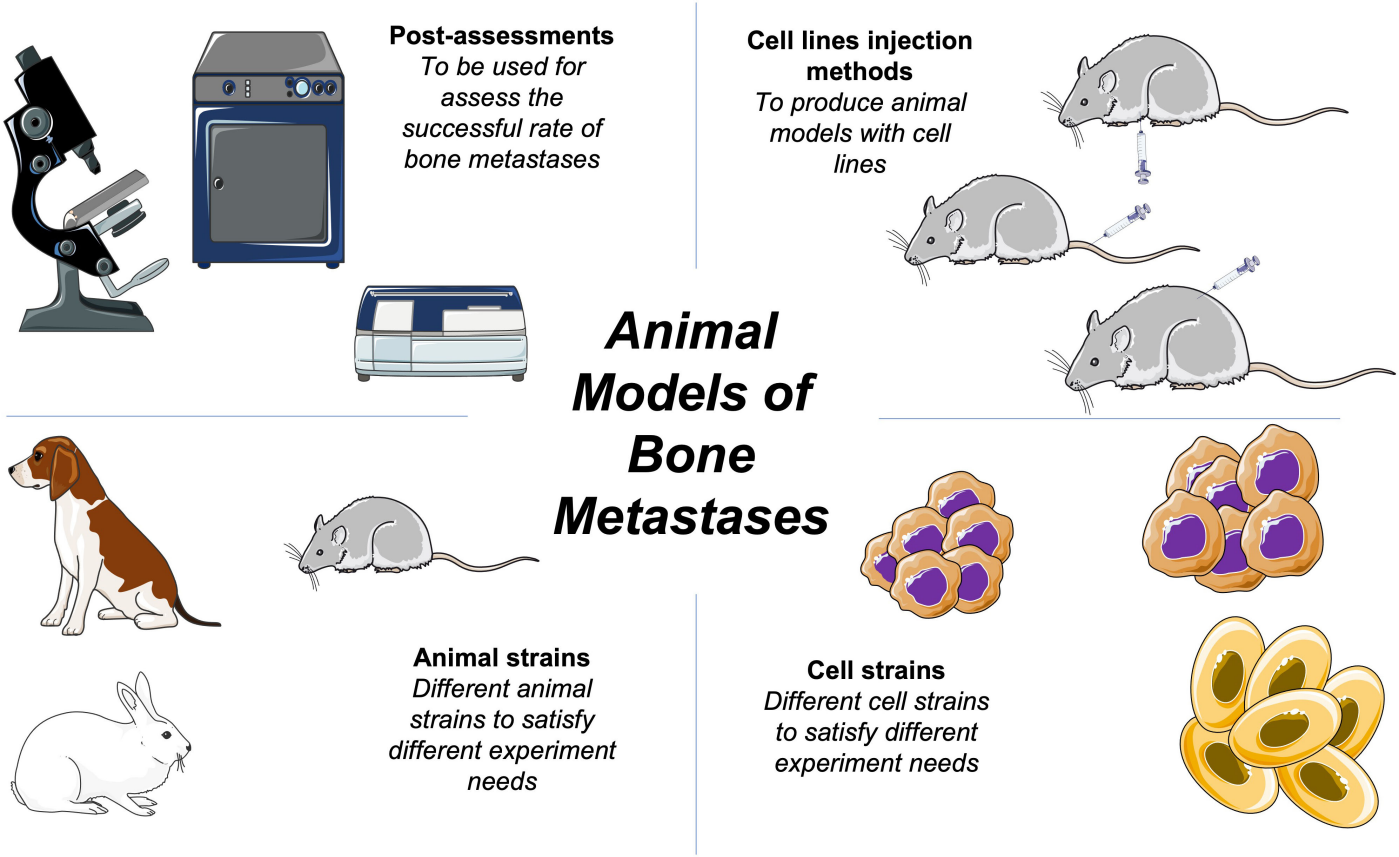
Schematic of basic bone metastases animal models methods.

In this review, we have discussed the animal models of bone metastasis most commonly used in preclinical experiments and their underlying mechanisms. No single model can represent all the genetic mechanisms of bone metastasis, which requires whole-body organisms. Here, we have compiled a selection of animal models to assist in future studies (**Figure 1**).

## 2 Commonly used animals in building animal models

Basing animal models of bone metastasis on general disease models is unreliable. Because the etiology of bone metastasis of human and animal cancers is different, different cancers have different metastatic targets. For example, mouse breast cancer may preferentially metastasize to the lung, while human breast cancer mainly metastasizes to the bone (2). Lung tumors may specifically metastasize to the vertebral column (8, 9). Hence, researchers are required to modify the animal models based on their experiments. The mouse is the most common animal of choice to construct bone metastasis models.

### 2.1 Breast cancer

Animal models based on human breast cancer cells are commonly constructed using rodents, such as mice or rats, and used in preclinical experiments (10). Both immunodeficient and immunocompetent animals are used. Nude mice of the Balb/c background are frequently used because they are susceptible to both human and rodent breast cancer cell lines (2). Due to the lack of a thymus, immune responses are hardly generated in most of these mice following the injection of cancer cells, which significantly improves the success rate of model construction. Non-obese diabetic/severe combined immunodeficiency (NOD/SCID) mice are immunodeficient mice commonly used in xenograft experiments. Disabilities in the immune system of NOD/SCID mice affect the growth of lymph cells as well as immune signaling. Yin’s team used NOD/SCID mice paired with the MDA-MB-231 cell line to investigate how runt-related transcription factor 2, an osteogenesis-related factor, promotes breast cancer and bone metastasis (11).

The demand for crossbred or genetically engineered mice has also increased to better meet experimental needs (12–16). Mice that have been crossed and repeatedly backcrossed can offer an *in vivo* environment better suited to investigate the mechanism of breast cancer bone metastasis (13). In Laura’s experiment, Col1a-Krm2 mice were backcrossed with NOD/SCID/IL2rγ^null^ (NSG) mice for 10 generations to introduce an immunocompromised background (13). They found that cancer metastasis to other organs like the spine may be prevented in rather young animals. By modifying the animal model into adult mice and backcrossing over 10 generations, they could focus on the early stages of human breast cancer metastasis. Devignes’ team also backcrossed Floxed mice bred in previous experiments with FVB/n wild-type mice for 10 generations to achieve genetic reconstitution consistent with their experimental requirements. Based on whether the *HIF* gene was expressed, mice were divided into two groups to verify whether the HIF signaling pathway in osteoblasts could promote breast cancer cell invasion and bone metastasis (14).

Unlike these experiments, Mercatali’s team used zebrafish as a special model to study bone metastasis (17). Visualizing zebrafish embryos and easy genetic manipulation provide researchers with a new method of studying cancer progression.

### 2.2 Prostate cancer

The first model of prostate cancer – the Dunning rat – exhibits a spontaneous development of the disease (7). However, this model did not show a tendency for bone metastasis, and R-3327 cells derived from the Dunning rat can only metastasize to the lymph nodes. Dogs are also listed as candidate animal models, but they rarely develop prostate cancer due to the lack of androgen receptors on their cell membranes (7). The internal organization of mice femur includes a high-woven bone structure that is less fibrolamellar in nature, providing conditions amenable for bone metastasis (10, 18).

Transgenic mouse models have the advantage of lacking immune responses to injected cells or xenografts (19). Transgenic adenocarcinoma of the mouse prostate (TRAMP) is one of the most famous transgenic models, exhibiting metastases to the lung and lymph nodes rather than the bone (19, 20). The promoters expressed in neuroendocrine cells, such as the probasin promoter in TRAMP, drive transgenic oncogene expression. NOD/SCID mouse is one of the most used immunodeficient animal models in prostate cancer bone metastasis experiments (21–25). Landgraf created a new model for studying prostate cancer bone metastasis by modifying NSG mice with a humanized tissue-engineered bone construct (hTEBC), which facilitates cancer cell growth (23). Ganguly’s team injected PC3 cells into the tibia of 6-week-old NSG mice to explore whether NOTCH3 induces tumor-specific elevation and secretion *via* MMP-3 (21).

However, the existing models are still limited to some of the detectable cancer-related factors, and cannot provide a comprehensive or linear picture of bone metastasis.

## 3 Cancer cell lines

Both patient-derived cancer tissues and immortalized cancer cell lines are used for transplantation. Patient-derived cancer tissues show genetic concordance between the clinic and the animal models, and help to establish consistent animal models specific to particular cancer cell lines. However, these models may face obstacles in the form of ethics and tissue availability. Cell lines, after several passages, can generate stable primary or secondary cancer sites. Moreover, researchers can genetically edit cell lines by using luciferase genes or knocking out certain genes (26–28).

### 3.1 Breast cancer

Immortalized human breast cancer cell lines, such as MDA-MB-231, 4T1, and MCF-7, are more easily available than patient-derived tissues. They possess obvious breast cancer target characteristics, and can also exhibit a tendency for bone metastasis after multiple passages (**Table 1**) (2, 5, 11, 51). They can help restore human bone metastasis in animal models. The bone-homing capabilities of MDA-MB-231 sub-lines can be enhanced *via* generation injections, and up to 90% of MDA-MB-231-bone cells can form neoplasms (52–54). Using 5–8-week-old mice is vital to achieve bone metastasis *via* intracardiac, intra-arterial, or intravenous injections. Farhoodi injected 4T1 cells into the mammary fat pad of Balb/c mice, and then examined their legs for bone metastases. Once its incidence was confirmed, the mice were sacrificed to collect the metastatic tumor cells from the leg bones. These cells were cultivated to purify tumor cells with bone-metastatic tendencies (51). They purified their experimental cells to improve the success rate.

**Table 1:** Common cancer cell lines in bone metastases.

Cancer	Cell Lines	Origin	Model System	Metastases Preference
BCa	MDA-MB-231	Human mammary adenocarcinoma from a 51-year-old Caucasian female	Balb/c nude, MF1 nude, NSG	Mouse long bones, spine and jaw (29–34)
	MCF-7	Human mammary adenocarcinoma from a 69-year-old Caucasian female	Balb/c nude, NOD/SCID	Mouse long bones (32–34)
	T47D	Human mammary ductal carcinoma isolated from a pleural effusion	Balb/c nude, NOD/SCID	Mouse long bones (35, 36)
	4T1	Stage IV mammary tumor from a female Balb/c cfC3H mouse	Balb/c cfC3H	Mouse long bones, Spine, jaw, lungs, and spleen (37–40)
PCa	PC3	Bone metastases from a 62-year-old white man	Balb/c nude, NOD/SCID, NSG	Mouse long bones, spine (33, 41–45)
	LNCaP	Supraclavicular lymph node from a 50-year-old white man	Balb/c nude, SCID	Mouse long bones, spine (29, 46–48)
	DU145	Brain metastases from a 69-year-old white man	Balb/c nude, Ncr nu/nu, NOD	Mouse long bones (25, 45, 47, 49, 50)

BCa, breast cancer; PCa, prostate cancer.

Different pairs of cell lines can also be combined to test certain concepts. Yin’s team compared MCF-7 and HCC1954 to validate whether KRT13, a protein from the keratin family, promotes stemness, metastasis, and cellular invasiveness (55). Han’s group estimated the metastatic rate of different cell lines (56). They found that the proliferation of MDA-MB-453, UACC-893, and HCC-202 cells increased in the eighth week, while MDA-MB-361, UACC-812, BT-474, and ZR-75-1 cells exhibited moderate proliferation but obvious migration. Using HCC-2218 and HCC1419 cells, tumors did not form, suggesting that both lack the ability to metastasize to the bone. The tumors formed by HCC-202 and MDA-MB-361 cells decreased in size after the sixth week, indicating that these two cell lines may not survive long-term metastasis (56). Eckhardt et al. also tested several cell lines, and NSG mice were used in xenograft studies involving MDA-MB-231 and SUM159 cells (37).

### 3.2 Prostate cancer

Like other cancer cell lines, those of prostate cancer also originate from both humans and animals (**Table 1**). R-3327, derived from the Dunning rat, has been used to investigate human prostate cancer due to its spontaneous neoplasm development (57). Other animal-derived cell lines, such as PA-III or AT6-1, naturally form osteolytic and osteoblastic lesions similar to human bone metastases in animal models (57–59). RM1, derived from the mouse prostate, is a highly metastatic cell line, but does not metastasize to the bone (60). Although it can induce consistent bone lesions in mouse models, it is a transformed cell line, not a natural one.

PC3, DU145, and LNCaP are patient-derived cell lines commonly used in prostate cancer animal models. They are easily available and possess the basic prostate cancer cell targets. PC3, derived from the bone metastases of a 62-year-old white man, was selected by isolating highly invasive cells from bone metastatic lesions. Landgraf implanted an hTEBC structure based on the bone-homing properties of PC3 cells, followed by an intracardiac injection of Luc-transfected cancer cells, facilitating the construction of models for transferring the human osteoblast line PC3 to hTEBC and the murine femur (23). Studies on LNCaP, PC3, and DU145 cells, all of which differ in their sensitivity to androgens, showed that prostate cancer-secreted growth differentiation factor 15 modulates the potential for bone remodeling in metastatic bone lesions (49, 61). Lang’s team grouped five common prostate cancer cell lines to verify whether PCAT7, a bone metastasis-related long non-coding RNA, activates the transforming growth factor-β/suppressor of mothers against decapentaplegic signaling pathway by upregulating transforming growth factor-β receptor 1. Its negative correlation with miR-324-5p was also investigated (62). Sohn’s team tried to intracardiacally inject LNCaP cell lines grouped with CD133^+^. The overexpression of CD133^+^ in LNCaP cells enhanced their cancer stem cell-like characteristics in terms of colony formation, migration, etc. The CD133^+^ group exhibited a bone metastasis rate of 80%, compared with 20% in the Vec group. Moreover, the CD133^+^ group showed a significant violation of the diffuse osteolytic characteristics of the spinal cord and the vertebral bodies (29).

## 4 Preparation of cell lines for transplantation

### 4.1 Orthotopic inoculation of cells

*In situ* injection of cancer cells best reproduces the process of cancer metastasis in the human body. Injected into mouse mammary fat pads, tumor cells can be seeded through the vasculature towards the target organs – a method that achieves 40–60% of bone metastases in breast cancer animal models (63). To study the function of TIE2, a tyrosine kinase receptor, in osteolytic bone metastasis, Drescher’s team administered both bilateral mammary fat pad injections and left ventricular injections to the grouped mice. The correlation between carcinoma *in situ* and bone metastasis was evaluated to determine whether TIE2 inhibition stimulates the dormant breast cancer cells and promotes bone metastasis (34). Likewise, Spadazzi’s team injected MCF-7 cells into the left ventricle and mammary fat pads of NSG mice to investigate whether trefoil factor-1 could exert estrogen-induced effects (64).

However, this method suffers from a considerable variation in metastatic tumor growth, besides the comorbidity caused by development of the tumor (**Table 2**) (73). In addition, it poses the problem of small bone metastases while the primary tumor has grown beyond an ethically reasonable size (5), which seriously compromises the detection of stimulated bone metastases.

**Table 2:** Implantation methods for bone metastases models.

Cell Injection Methods	Module of metastases studied	Advantages	Disadvantages
Orthotopic Inoculation	Primary tumor and invasively distant metastases	Study of tumor growth *in situ* and distant metastases	Unstable bone metastasis success rate (65–67)
Intracardiac	Circulation and metastases	Easily producing metastases	Requiring sophisticated skills (68–70)
Caudal Vessels	Circulation and metastases	More visualization of circulation inoculation	Potential lung metastases (7, 24, 51)
Intraosseous	Bone metastases	Most convenient and successful method for bone metastases models	Not reflecting the complete course of tumor metastasis (71)
Allografts/Xenografts	Depend on location	Reflecting natural heritability and cellular heterogeneity	Usually requiring immunodeficient mice and high maintenance (23, 72)

Some scientists have also suggested subcutaneous allografts to model bone metastasis. Peiffer’s team provided a detailed protocol of resecting subcutaneous prostate cancer allografts from immunocompetent mice (65). Bone metastases, abdominal cavity metastases, and local invasion all occurred in eight mice. This study demonstrated that resection of subcutaneous allografts from mice can lead to the development of metastasis; however, the duration of the experiment was extended by the removal of the prostate gland and precise operations.

### 4.2 Intravascular injection

Intravascular injection is a way of inoculating cells into the blood circulation. Unlike in orthotopic or ectopic inoculation, tumor cells injected *via* this method can localize to the target site through the intravascular circulation (**Table 2**) (66). Intra-arterial injections are usually administered to the left ventricle, limiting the clearance of cells that occurs when they pass through the lung capillaries (10, 53, 67). Tail vein injection, which is the more common intravenous injection today, effectively increases the rate of bone metastasis while also increasing the rate of mortality in mice (51).

Animal models currently rely on intracardiac injections to realize the process of bone metastasis. Tumor cells are injected into the circulation through the left ventricle of mice, after which they go through the processes of adhesion, degradation, and migration to finally cause metastases in different organs, thereby simulating the process of bloodway metastasis of tumors. Using intracardiac injections to probe the role of cancer-associated factors in the regulation of tumor bone metastasis has become the preferred modeling approach (44–46). Zheng et al. used this method to prove that osteoblastic Niche-derived Jagged1 sensitizes bone metastases (15). Wang’s team showed that the bone sialoprotein–αvβ3 integrin axis functioned significantly more efficiently in cancer cell bone metastasis when integrin was overexpressed. For comparison, stained specimens of the brain, lung, tibia, and femur were collected after left ventricular injection in nude mice (52). Although the postoperative mortality is relatively high, the survival rate can still exceed 90% with practice.

Caudal vessel injection can produce a higher rate of metastasis to the leg bone than to other vital organs. This method offers better accuracy than intracardiac injection because the visibility of tail vessels enables researchers to observe the flow of cancer cell fluids within (74). Caudal vascular injections can either be intravenous or arterial. Injecting through the tail artery will reduce the elimination of tumor cells in pulmonary capillaries and improve the success rate of colonization to the bone, while tail vein injection will promote tumor metastasis to the lung (2, 51, 74). In Farhoodi’s experiments, the 4T1 cell model tail artery injection mice showed a significant number of tumor cells localized to the subinguinal fat pad and the leg bone (51). Tumor cells were found in the leg bones of all 32 mice injected through the tail artery, and the rate of bone metastasis following complete tail veil injection was greater than 90% as well. Metastases were also detected in 70% of other target locations 2 weeks post-injection. Hamaidi et al. determined the effect of Lim1 on the adhesion, epithelial–mesenchymal transition, invasion, and metastatic progression of cancer cell surface targets after injection of the renal carcinoma cell line Caki2/786 through the lateral caudal vein of nude mice (75). However, caudal vein injection also resulted in metastatic foci in the lungs of mice.

Multiple factors affect the success of experiments involving vascular injection. Operator skill gaps, standard cell operation procedures, and pressure within the caudal vessels can all influence the growth rate and success of tumor bone metastasis (51). Dilation of the caudal vessels prior to injection or the use of fluorescein to reveal vessel flow can improve the effectiveness of the injection. Non-directed intracardiac injection is still associated with a risk of thrombosis due to the procoagulant activity of tumor cells after accurate completion. The mortality of post-inoculation animal models may be reduced by injecting low-molecular weight heparin into the tail vein 10 minutes before inoculation (76).

### 4.3 Intraosseous injection

Metastatic tumors can bypass the pre-metastatic process if they are directly ectopically implanted into the bone. The growth of tumor cells inside the bone depends on their interaction with bone cells and the bone microenvironment (**Table 2**) (77, 78). Therefore, while intraosseous injection can help examine local tumor behavior within the bone microenvironment, it cannot be used to study the early stages of bone metastasis (79). Researchers typically inject 50,000–100,000 cancer cells directly into the tibia or femurs of mice, avoiding the possible comorbidity of the animals’ primary tumor (80, 81). Chen et al. observed that Brachyury, one gene affects tail length in mice, was expressed at a low level in the highly metastatic MDA-MB-231 cell line while it was highly expressed in the poorly metastatic T47D cell line when breast cancer cells were injected into the top anterior condylar region of the right tibia of mice. Nude mice showed significant swelling at the injection site 4 weeks post-injection, and X-ray revealed tumor-induced osteolytic lesions (35). After injecting prostate cancer cells into the left tibia of Balb/c nude mice, Thulin’s team performed bone tumor development status assays using peripheral quantitative computed tomography (CT) and microCT to investigate the effect of signal transducer and activator of transcription 3 (STAT3) inhibitors on STAT3-regulated prostate cancer bone metastasis. The STAT3 inhibitor treatment resulted in an intact tibial bone microenvironment with no tumor formation or sclerotic response in mice, whereas the VCaP group showed sclerotic bone tumor response up to 85% (48).

### 4.4 Allograft and xenograft models

Transplanting allogeneic or xenogeneic tissues into animal models is a common way of modeling bone metastasis (**Table 2**). Since animals with different genetic backgrounds respond to allogeneic tissues differently, selecting the appropriate tissue source is especially important. In the case of xenografts, patient-derived tumor tissues can better reflect the biological characteristics of tumor bone metastasis in humans (82). Patient-derived xenografts aim to directly transplant human tumor tissue into immunodeficient mice, which represents natural heritability and cellular heterogeneity in human cancer better than simple cell-transplantation models (83). Among animal models, xenografts can only be performed in immunocompromised or immunodeficient animals. Aoki et al. first grew tumor tissue from bone metastases by intraperitoneally injecting it into male thymus-free nu/nu nude mice (42). The tumors were surgically processed to 1-mm^3^ fragments to be implanted into the proximal left tibia of the nude mice when they reached 10 mm in diameter. They observed tumor growth in all eight mice. Landgraf’s hTEBC model is likewise based on the low immune response of NSG mice to xenografts, while adding humanized components to mimic human tumor bone metastasis as satisfyingly as possible in mice (23).

## 5 Assessment of animal models of bone metastasis

After injecting cancer cells into mice, bone lesions develop quickly, necessitating researchers to detect physiological conditions, bone changes, and tumor lesions in a timely manner.

Establishing bone metastasis models using luciferase or fluorescent protein-labeled cell lines allows researchers to monitor tumor development in the bones of living animals (15, 39–41). Oliemuller et al. studied the effects of SOX11 on cell invasion and bone metastasis using DCIS-Luc cells, generated by transducing the cells with luciferase 2 lentiviral particles (84). Arriaga’s team bred NPK^EYFP^ mice by crossing NPK mice with the Rosa-CAG-LSL-EYFP-WPRE reporter allele, facilitating *in vivo* fluorescence visualization and quantification of YFP-positive prostate tumors and metastases (85).

In turn, instrumentation such as the IVIS system can provide more accurate quantitative indicators through fluorescent or bioluminescent readings obtained from tumors (76–78). Typically, tumor growth in the bone is measured once or twice a week. The area of osteolytic lesions and abnormal bone remodeling can be assessed visually by X-ray or *in vivo* microCT (45–47, 85). Hinz’s team then used the IVIS system. After injecting MDA-MB-231 cells into the left ventricle of NSG mice, they performed IVIS bioluminescence assays weekly to assess osteolytic lesions caused by bone metastasis from triple-negative breast cancer. The inoculation of AKT3-knockout 231-BO cells into NSG mice resulted in enhanced bone metastases (86). Another team validated the effect of intracardiacally injecting MDA-MB-231-derived osteotropic cells into nude mice by examining osteolytic lesions in their hind tibia and femurs by microCT. MicroCT images showed that NKX2-8-silenced cell lines were more likely to produce earlier bone metastases, while its overexpression delayed the appearance of metastases, inhibited osteoclast activity, and reduced bone metastatic lesions (87).

At the end of the animal test, the mice should be examined simultaneously for extraosseous metastases. All relevant organs and metastases are fixed in 10% formalin for analysis. For histological studies, samples are fixed in paraformaldehyde for 24–48 hours and then decalcified in paraformaldehyde/ethylenediaminetetraacetic acid solution for 2 weeks. The decalcified paraffin-embedded bone should be sectioned for hematoxylin and eosin staining and evaluated using image analysis software. Bone conversion-related growth factors in the serum can also be assayed (88, 89). Metastases from the lung, liver, and brain tissue can likewise be analyzed and studies investigating the correlation between the area and the number of bone metastases can be performed (90).

## 6 Conclusion

Bone metastasis is a common manifestation of cancer deterioration in the mid and late stages of the disease. Much research has been done on the invasion of cancer cells, from migration to the bone tissue and beyond; however, much needs to be understood yet. Animal models are vital tools in preclinical metastatic experiments that can help identify the key steps in bone metastasis. Here, we have summarized the experimental animals, cell lines, cell implantation techniques, and evaluation methods used while studying common breast and prostate cancer bone metastases. For preclinical animal testing, immunodeficient animals are used to achieve xenograft growth without eliciting a host immune response. In preclinical studies, many investigators have successfully improved the success of tumor cell colonization to the bone by backcrossing cell lines and transgenic mice. More importantly, most animal tests related to cancer bone metastasis have been performed using cancer cell line injection models. Although the early stages of bone metastasis cannot be studied, these models are effective for studying the interaction between cancer cells and the bone microenvironment.

However, using mice to study human tumor immunity has its limitations. The differences in bone metastasis pathways between humans and animal models can explain why the success of preclinical treatments is not perfectly reproduced in humans. The inability to present a complete and comprehensive picture of the whole process of bone metastasis is also a problem that needs to be addressed while engineering animal models today.

## Author contributions

All authors contributed equally to this work. All authors contributed to the article and approved the submitted version.
